# A *k*-nearest neighbor classification of hERG K^+^ channel blockers

**DOI:** 10.1007/s10822-016-9898-z

**Published:** 2016-02-10

**Authors:** Swapnil Chavan, Ahmed Abdelaziz, Jesper G. Wiklander, Ian A. Nicholls

**Affiliations:** Bioorganic and Biophysical Chemistry Laboratory, Department of Chemistry and Biomedical Sciences, Linnaeus University Centre for Biomaterials Chemistry, Linnaeus University, 391 82 Kalmar, Sweden; eADMET GmbH, Lichtenbergstraße 8, 85748 Garching, Munich, Germany; Department of Chemistry-BMC, Uppsala University, Box 576, 751 23 Uppsala, Sweden

**Keywords:** Classification model, hERG blockers, Ikr, KCNH2, *k*-nearest neighbor (*k*-NN), Toxicity

## Abstract

A series of 172 molecular structures that block the hERG K^+^ channel were used to develop a classification model where, initially, eight types of PaDEL fingerprints were used for *k*-nearest neighbor model development. A consensus model constructed using Extended-CDK, PubChem and Substructure count fingerprint-based models was found to be a robust predictor of hERG activity. This consensus model demonstrated sensitivity and specificity values of 0.78 and 0.61 for the internal dataset compounds and 0.63 and 0.54 for the external (PubChem) dataset compounds, respectively. This model has identified the highest number of true positives (i.e. 140) from the PubChem dataset so far, as compared to other published models, and can potentially serve as a basis for the prediction of hERG active compounds. Validating this model against FDA-withdrawn substances indicated that it may even be useful for differentiating between mechanisms underlying QT prolongation.

**Electronic supplementary material:**

The online version of this article (doi:10.1007/s10822-016-9898-z) contains supplementary material, which is available to authorized users.

## Introduction

The human ether-a-go-go related gene (hERG, KCNH2) encodes for a voltage dependent K^+^ ion channel (Kv11.1). Blocking of this channel has been associated with potential severe heart arrhythmia, and because of this, several drugs have been withdrawn from the market [1–6]. Further, the drug-induced long QT syndrome may cause avoidable sudden cardiac arrest [3, 4]. With the intention of protecting clinical trial participants and patients, the International Conference of Harmonization published a guideline (S7B) recommending that “all new drugs” should be tested pre-clinically for hERG sensitivity and cardiac safety before submitting an application to regulatory reviews [7]. Accordingly, the early assessment of hERG-related cardiotoxicity has become a common practice in drug discovery.

Many in vitro assays exist for the pre-clinical evaluation of hERG-related cardiotoxicity [8], examples include rubidium-flux assays, radioligand binding assays, in vitro electrophysiology measurements, and fluorescence-based assays [9]. In addition, in silico models have been proposed for identifying potential hERG blockers in drug discovery processes [10, 11].

Efforts to use computational methods for the prediction of hERG blocking effects have ranged from the use of simple rules based on structural and functional features, through to more complex quantitative structure–activity relationship (QSAR) models [12–16]. A number of QSAR models have been developed for the hERG toxicity endpoint using different machine learning algorithms, such as multiple linear regressions [17], partial least squares (PLS) [18], *k*-nearest neighbor algorithms (*k*-NN) [19], artificial neural networks [20], support vector machines (SVM) [21], random forest [22] and naive Bayesian classifications [23]. Despite these efforts there is significant scope for development of more powerful and more easily deployed predictive models.

The recent development of open source fingerprints, such as PaDEL fingerprints, which are libraries of descriptors [24], allows for ready access to tools for predicting biological endpoints. A recent report on the use of PaDEL fingerprints in conjunction with a *k*-NN strategy aimed at the prediction of chronic toxicity [25] prompted us to apply this approach to hERG-channel blockers, a far more focused system. It was envisaged that publicly available data on a series of hERG-channel blockers could function as a starting point for model construction, and a series of 1953 PubChem compounds could act as basis for validation.

## Methodology

### Description of dataset

IC_50_ data for 172 I_kr_ (‘rapid’ delayed rectifier current) channel blockers were retrieved from the webservers OCHEM [26] and Fenichel [27]. These 172 compounds are structurally diverse and belong to different therapeutic classes. The compounds were authenticated with respect to structure and IUPAC name. After authentication, the SMILES notations for all the 172 compounds were verified using ChemSpider [28], SigmaAldrich [29] and PubChem [30]. A PubChem dataset comprised of 1953 entries was chosen for the external validation [31]. Dataset entries that were mixtures or salts were discarded, leading to a final PubChem validation set of 1795 compounds. More details about the training and test set compounds are provided in the Online Resources 1 and 2, respectively.

### Descriptor calculation

The descriptor calculation was a primary requirement for the construction of the classification model. Eight types of PaDEL fingerprints were calculated for both the training and test set compounds using PaDEL software [24]. These consisted of the CDK, Extended CDK, CDK Graph, Estate, MACCS, PubChem, Sub-structure and Sub-structure count fingerprints. Each of the eight types of fingerprints was then used, separately, to develop a classification model.

### Class assignment

The training set compounds were split into one of the two classes (active and inactive) using an IC_50_ threshold value of 5 µM. The PubChem dataset derived test set compounds were similarly classified, i.e. as either active or inactive, here using a % inhibition threshold of 20 %. A summary of the numbers of the compounds and their classes is provided in Table 1.

**Table 1:** Classification of training and test set compounds

	Class 1 (hERG active)	Class 2 (hERG inactive)	Total
Training	93	79	172
Test	221	1574	1795

### Software and modules

The Matlab module “classification_toolbox” [32] was employed for the development of the *k*-NN classification model. The Matlab module is freely available at [33].

### Classification model development

The *k*-nearest neighbor (*k*-NN) classification method employed used cross validation (CV) to identify optimal *k* values [34, 35]. A series of *k* values (from 1 to 10) were assigned to construct the model, and by determining the lowest class error, optimal *k* values were identified.

A five-step cross validation was implemented by first dividing the training set into five equal groups, four of which were used for model construction and the remaining for validation. This procedure was repeated so that each of the five groups was used for validating the models constructed using the remaining four. After cross validation, the models were subjected to external validation using the 1795 PubChem compounds. The performance of each classification model was assessed by means of statistical parameters, such as non-error rate (NER), sensitivity, specificity, precision and error rate [36]. The models were then analysed and compared on the basis of these statistical parameters.

## Results and discussion

### Construction of eight *k*-NN classification models

The *k*-nearest neighbor (*k*-NN) classification method was employed to construct classification models using each of the eight PaDEL fingerprints. Employing the *k*-NN algorithm requires that the optimal value of *k* is determined [34]. There are several ways to determine the *k* value, e.g. through application of a risk function or empirical rules, or through cross validation. Here, cross validation was used to determine the optimal *k* value.

A series of eight *k*-NN classification models was constructed using each of the PaDEL fingerprints, and compared with respect to a series of statistical parameters, Table 2.

**Table 2:** Summary of statistical parameters for the *k*-NN classification models

Entry	Fingerprints	NER	*k*	Sensitivity		Specificity	
				Class 1	Class 2	Class 1	Class 2
1	*CDK*						
	Fitting	0.68	1	0.72	0.65	0.65	0.72
	CV	0.66	1	0.72	0.61	0.61	0.72
	External	0.54	1	0.52	0.57	0.57	0.52
2	*Estate*						
	Fitting	0.68	1	0.73	0.62	0.62	0.73
	CV	0.66	1	0.72	0.61	0.61	0.72
	External	0.53	1	0.49	0.57	0.57	0.49
3	*Extended CDK*						
	Fitting	0.67	1	0.70	0.63	0.63	0.70
	CV	0.65	1	0.70	0.61	0.61	0.70
	External	0.56	1	0.56	0.57	0.57	0.56
4	*CDK graph*						
	Fitting	0.64	1	0.69	0.59	0.59	0.69
	CV	0.64	1	0.70	0.58	0.58	0.70
	External	0.55	1	0.52	0.57	0.57	0.52
5	*MACCS*						
	Fitting	0.68	6	0.76	0.59	0.59	0.76
	CV	0.67	6	0.76	0.57	0.57	0.76
	External	0.55	6	0.54	0.55	0.55	0.54
6	*PubChem*						
	Fitting	0.60	3	0.69	0.52	0.52	0.69
	CV	0.60	3	0.71	0.49	0.49	0.71
	External	0.57	3	0.62	0.52	0.52	0.62
7	*Sub*-*structure*						
	Fitting	0.68	1	0.70	0.67	0.67	0.70
	CV	0.67	1	0.69	0.66	0.66	0.69
	External	0.57	1	0.54	0.59	0.59	0.54
8	*Sub*-*structure count*						
	Fitting	0.67	1	0.74	0.61	0.61	0.74
	CV	0.68	1	0.72	0.65	0.65	0.72
	External	0.58	1	0.61	0.56	0.56	0.61

CDK fingerprints are one-dimensional 1024 bit long arrays that are arranged based upon the occurrence of particular structural elements. The Extended CDK fingerprints are extended versions of CDK fingerprints that include ring features. Graph fingerprints are specialized versions of the CDK fingerprints that exclude bond orders. Estate fingerprints represent the influence of substituent electronic effects in a given compound. PubChem fingerprints are binary substructure fingerprints of length 881. MACCS fingerprints consist of 166 keys that are based on SMARTS patterns [37, 38]. The Sub-structure fingerprints represent 307 SMARTS patterns for different functional groups, whereas the count of these SMARTS patterns is referred to as the Sub-structure count fingerprint [37].

The sensitivity expresses the prediction accuracy of hERG-active compounds, whereas specificity reflects the prediction accuracy for hERG-inactive compounds. The models performed similarly in terms of the statistical parameters examined. Thus, to further improve the predictive power of these models we developed a series of consensus models. Several methods have been reported for consensus model development [39]. For classification models, the majority principle [40] is commonly employed and we have used this strategy to develop consensus models based upon three, five and seven different fingerprint-based models. As it is more important to identify hERG-active compounds than hERG-inactive compounds, the eight models (from Table 2) were examined with respect to their sensitivity in the external prediction. The Estate-fingerprint-based model exhibited relatively poor sensitivity (0.49) and was discarded from the consensus model building procedure to provide an odd number (seven) of fingerprints. Six consensus models were built using different combinations of the seven remaining fingerprint-based models, Table 3.

**Table 3:** Statistical parameters for the consensus models

Model^a^	Dataset	TP^b^	FP^c^	TN^d^	FN^e^	TP + TN	Total^f^	Q^g^	Sens.^h^	Spec.^i^	Prec.^j^	G-mean^k^
1	Training	72	25	54	21	126	172	0.73	0.77	0.68	0.74	0.73
	Validation	130	654	920	91	1050	1795	0.58	0.59	0.58	0.17	0.59
2	Training	73	31	48	20	121	172	0.70	0.78	0.61	0.70	0.69
	Validation	140	723	851	81	991	1795	0.55	0.63	0.54	0.16	0.59
3	Training	71	31	48	22	119	172	0.69	0.76	0.61	0.70	0.68
	Validation	135	707	867	86	1002	1795	0.56	0.61	0.55	0.16	0.58
4	Training	74	32	47	19	121	172	0.70	0.80	0.59	0.70	0.69
	Validation	128	718	856	93	984	1795	0.55	0.58	0.54	0.15	0.56
5	Training	73	29	50	20	123	172	0.72	0.78	0.63	0.72	0.70
	Validation	132	685	889	89	1021	1795	0.57	0.60	0.56	0.16	0.58
6	Training	73	28	51	20	124	172	0.72	0.78	0.65	0.72	0.71
	Validation	131	675	899	90	1030	1795	0.57	0.59	0.57	0.16	0.58

^a^Model 1 = substructure (SS) + substructure count (SSC) + extended CDK (ECDK), 2 = PubChem (PC) + SSC + ECDK, 3 = PC + SSC + SS, 4 = PC + SSC + MACCS, 5 = PC + SSC + ECDK + SC + MACCS, 6 = PC + SSC + ECDK + SS + MACCS + CDK + CDK Graph, ^b^ true positives, ^c^ false positives, ^d^ true negatives, ^e^ false negatives, ^f^ TP + TN + FP + FN, ^g^ overall accuracy of prediction, ^h^ sensitivity, ^i^ specificity, ^j^ precision, ^k^  $\sqrt {{\text{Sensitivity}} \times {\text{Specificity}}}$

Although consensus model 1 shows better overall accuracy of prediction (Q), consensus model 2 shows higher sensitivity for test set prediction, and was thus chosen for further studies.

### Individual contribution of each model

**Fig. 1:**
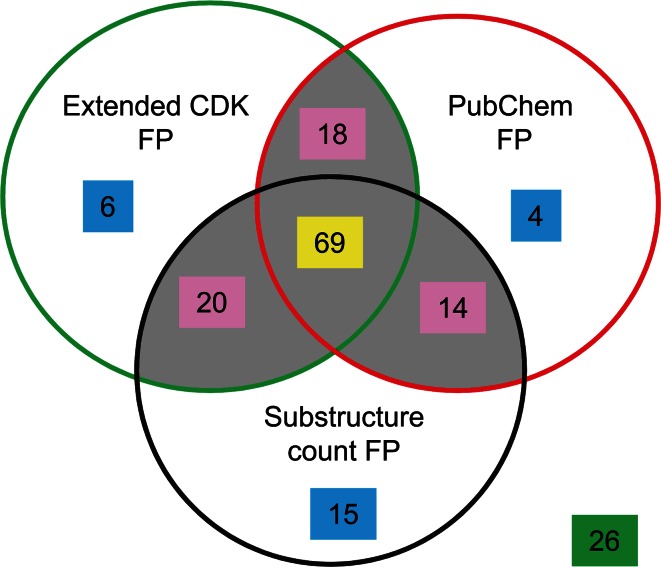
Venn diagram representing the number of training set compounds correctly predicted by all three models (*yellow*), by any two models (*magenta*), by only one model (*blue*) and by none of the models (*green*). The *shaded area* represents compounds correctly predicted by the consensus model

With consensus model 2 in hand, we then examined how individual training set compounds were handled by the consensus model as well as the individual models, i.e. Extended CDK, PubChem and Substructure count fingerprint based, Fig. 1.

The consensus model correctly predicted 121 of the 172 training set compounds. 69 of these 121 compounds were predicted correctly by all three individual models, while the remaining 52 compounds were correctly predicted by any two of the three models. Conversely, the consensus model incorrectly predicted 51 training set compounds. Of these 51, 25 compounds were predicted correctly by any one of the three models, whereas the remaining 26 compounds were incorrectly predicted by all three models.

In the case of the Extended fingerprint based model, 113 of 172 compounds were correctly predicted, 65 of which were hERG actives. The PubChem fingerprint based model predicted 105 compounds correctly from the training set. Among the 105 correctly predicted compounds, 66 were from class 1 and 39 from class 2. The Substructure count fingerprint based model predicted 118 training set compounds correctly. These 118 compounds were comprised of 67 compounds from class 1 and 51 compounds from class 2.

Compounds for which activities were not correctly predicted by our models are of interest as awareness of factors contributing to the incorrect prediction of compounds can help in the refinement of models. In this case, the IC_50_ value-based endpoints are derived from a range of studies so impact of inter-laboratory variation in the reported IC_50_ data on model performance cannot be excluded.

### Comparison of our model with other models

External validation provides an assessment of the QSAR model’s performance, and to compare models it is necessary that the external validations are performed on the same dataset. The PubChem dataset is comprised of 221 hERG-actives and 1574 hERG-inactives. Sensitivity and specificity are generally used to assess classification performance in imbalanced binary class studies [41]. G-mean, which is a geometric mean of sensitivity and specificity, was also used to measure the performance of the classification method in predicting actives and inactives. In studies aimed at the effective detection of only one class, as in our case where the prediction of hERG-actives is a priority, sensitivity and F-measures are often adopted [41]. Accordingly, we have compared our model with previously published models that were externally validated with the PubChem dataset [18, 42–44], with respect to sensitivity, specificity, G-mean and F-measure, Table 4.

**Table 4:** Comparison of the *k*-NN classification model with other models

Model	Our study	Su et al. [42]	Wang et al. [43]	Su et al. [18]	Li et al. [44]
Method	*k*-NN	SVM	Naive Bayesian classifier	PLS transformed into binary QSAR	SVM
Descriptors	2D PaDEL fingerprints	2D and 3D MOE, 4D fingerprints from MD simulation	Physico-chemical property based and geometry based descriptors, and fingerprints	2D and 3D MOE descriptors and 4D fingerprints	GRIND descriptors derived from docking
*Training set*					
Cut-off (µM)	5	–	10	40	40
Total	172	546	719	250	495
True positives	73	188	247	–	83
True negatives	48	242	315	–	283
Sensitivity	0.78	0.90	0.89	–	0.55
Specificity	0.61	0.72	0.72	–	0.83
Q	0.70	0.79	0.78	–	0.74
F-measure^a^	0.74	0.76	0.76	–	0.56
G-mean	0.69	0.80	0.80	–	0.67
*Test set*					
Cut-off (%)^b^	20	20	20	20	20
Total	1795	1668	1953	1668	1877
True positives	140	67	135	121	107
True negatives	851	1298	1247	963	1271
Sensitivity	0.63	0.41	0.54	0.74	0.57
Specificity	0.54	0.86	0.73	0.64	0.75
Q	0.55	0.82	0.71	0.65	0.73
F-measure	0.26	0.31	0.32	0.29	0.30
G-mean	0.59	0.60	0.63	0.69	0.66

^a^2[(precision*sensitivity)/(precision + sensitivity)], ^b^ % hERG blockage

As presented in Table 4, three of the four previously described models demonstrate lower overall sensitivities than our model, though it should be pointed out that IC_50_ thresholds used in the various studies varied between 5 and 40 µM. From a drug development perspective, it may be argued that it is of more interest to identify the potent hERG blockers (class 1) than hERG inactive compounds (class 2). Comparison on this point reveals that our model demonstrates better performance in predicting the hERG active compounds (True positives = 140, Sensitivity = 0.63) than the other models except that of Su et al. [18] in their model presented 2010. There, 163 hERG actives from the PubChem dataset were used for the external validation, whereas in our study a somewhat more comprehensive external validation was performed using 221 hERG actives.

From a practical perspective, ease of use is an issue of importance and an advantage of our model is that PaDEL fingerprints are fast and easy to calculate and do not involve complicated descriptor selection procedures. This is in contrast with all the other models presented in Table 4 that all employed 3D and 4D descriptors that require geometry optimization, a task necessitating significant computational resources. In addition, the application of different descriptor selection procedures makes these tasks more cumbersome. Therefore, in comparison to the other models, our model has the advantage of being fast, simple and relatively efficient in predicting hERG toxic compounds.

To further assess the potential of our consensus model, we turned our attention to the series of 47 substances withdrawn from use on account of QT-prolongation, which can be hERG-derived, as present in the WITHDRAWN database [45] (database last updated December 2015). Our training set had included 32 of these 47 drugs (shown in bold in Online Resource 1) of which our model had correctly predicted the IC_50_-based classes of 22. We interrogated the remaining 15 withdrawn substances (see Online Resource 3) using our model, which correctly predicted the IC_50_-based classes of 11 (73 %, see Online Resource 4). It is important to note that our model is solely based upon in vitro data (hERG IC_50_), while the basis for withdrawal, QT prolongation, is in vivo data-derived. The interpretation of the QT prolongation endpoint is itself a major challenge as mechanisms other than hERG activity can also underlie QT prolongation [4, 46, 47]. This is reflected in the fact that substances were correctly classified as class 1 or class 2, five and six substances respectively, based on their hERG IC_50_. This observation suggests that the model may even be useful for differentiating between mechanisms underlying QT prolongation.

A general reflection upon examining the hERG active compounds predicted by our model was the prevalence of aromatic and basic functionalities in these compounds (for example, see Online Resource 2). These features have previously been identified as essential components in a pharmacophore for central nervous system activity [48, 49] and we believe should be considered in future model development. Moreover, this may be considered indicative of a common evolutionary origin for the hERG voltage dependent K^+^ ion channel and CNS receptors [50, 51].

## Conclusion

In conclusion, PaDEL fingerprint-based *k*-NN classification models presented here show potential as tools for the prediction of the hERG toxicity endpoint, an important issue in modern drug development. In particular, the consensus model developed using the Extended CDK, PubChem and Sub-structure count fingerprint-based models performed comparably with models employing more complicated descriptors in the validation with external datasets. Moreover, the model presented here, in terms of the prediction of hERG toxicity, compares most favorably with these previously published models. Moreover, validating this model against FDA-withdrawn substances indicates that the model may be useful for differentiating between hERG-derived QT prolongation and other QT prolongation mechanisms. Accordingly, we believe that this model may provide a basis for improved drug design.

## Electronic supplementary material

Supplementary material 1 (XLS 58 kb)

Supplementary material 2 (XLS 628 kb)

Supplementary material 3 (XLS 30 kb)

Supplementary material 4 (XLS 30 kb)

## Abbreviations

CDK: Chemistry development kit
CV: Cross validation
hERG: Human ether-a-go-go-related gene
IUPAC: International union of pure and applied chemistry
*k*-NN: *k*-nearest neighbor
MACCS: Molecular ACCess system
NER: Non-error rate
QSAR: Quantitative structure–activity relationship
SMARTS: SMILES arbitrary target specification
SMILES: Simplified molecular-input line-entry system
